# Poly[triaqua­tris­(μ_4_-pyridine-3,5-dicarboxyl­ato)dicerium(III)]

**DOI:** 10.1107/S1600536811000286

**Published:** 2011-01-15

**Authors:** Fwu Ming Shen, Shie Fu Lush

**Affiliations:** a Department of Biotechnology, Yuanpei University, HsinChu 30015, Taiwan; b Department of General Eduction Center, Yuanpei University, HsinChu 30015, Taiwan

## Abstract

The asymmetric unit of the title compound, [Ce_2_(C_7_H_3_NO_4_)_3_(H_2_O)_3_]_*n*_, contains two Ce^III^ cations, three pyridine-3,5-dicarboxyl­ate (pyd) anions and three coordinated water mol­ecules. One Ce^III^ cation is coordinated by seven carboxyl­ate O atoms from six pyd anions and two water mol­ecules in a square-face-capped square-anti­prismatic geometry. Another Ce^III^ cation is coordinated by seven O atoms from six pdy anions and one water mol­ecule in a bicapped trigonal–prismatic geometry. The pdy anions bridge the Ce^III^ cations, forming the three-dimensional polymeric structure. The crystal structure contains extensive O—H⋯O, O—H⋯N and weak C—H⋯O hydrogen bonds. π–π stacking is present in the crystal structure, the shortest centroid–centroid distance between parallel pyridine rings being 3.509 (4) Å.

## Related literature

3,5-PydH_2_ can be easily deprotonated to the *N*-donor multidentate anion (pyd^2−^), enabling the ligand to act as a bridge to 3*d* and/or 4*f* metal ions, see: Jia *et al.* (2006 ▶). For related structures, see: Guo *et al.* (2009 ▶); Li (2007 ▶); Yi *et al.* (2009 ▶).
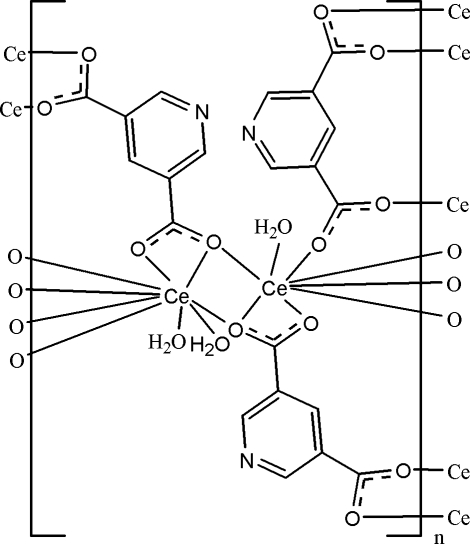


## Experimental

### 

#### Crystal data

[Ce_2_(C_7_H_3_NO_4_)_3_(H_2_O)_3_]
*M* *_r_* = 829.60
Triclinic, 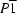

*a* = 8.959 (3) Å
*b* = 9.429 (3) Å
*c* = 14.582 (4) Å
α = 98.115 (6)°
β = 95.501 (6)°
γ = 105.030 (6)°
*V* = 1166.4 (6) Å^3^
*Z* = 2
Mo *K*α radiation
μ = 3.94 mm^−1^
*T* = 295 K
0.15 × 0.08 × 0.02 mm

#### Data collection

Bruker SMART CCD area-detector diffractometer
Absorption correction: multi-scan (*SADABS*; Bruker, 2001 ▶) *T*_min_ = 0.756, *T*_max_ = 0.975
12583 measured reflections
5586 independent reflections
2847 reflections with *I* > 2σ(*I*)
*R*_int_ = 0.090

#### Refinement

*R*[*F* ^2^ > 2σ(*F* ^2^)] = 0.041
*wR*(*F* ^2^) = 0.076
*S* = 0.74
5586 reflections
370 parameters
H-atom parameters not refined
Δρ_max_ = 1.36 e Å^−3^
Δρ_min_ = −1.15 e Å^−3^

### 

Data collection: *SMART* (Bruker, 2007 ▶); cell refinement: *SAINT* (Bruker, 2007 ▶); data reduction: *SAINT*; program(s) used to solve structure: *SHELXS97* (Sheldrick, 2008) ▶; program(s) used to refine structure: *SHELXL97* (Sheldrick, 2008) ▶; molecular graphics: *PLATON* (Spek, 2009 ▶); software used to prepare material for publication: *PLATON*.

## Supplementary Material

Crystal structure: contains datablocks global, I. DOI: 10.1107/S1600536811000286/xu5131sup1.cif

Structure factors: contains datablocks I. DOI: 10.1107/S1600536811000286/xu5131Isup2.hkl

Additional supplementary materials:  crystallographic information; 3D view; checkCIF report

## Figures and Tables

**Table 1:** Selected bond lengths (Å)

Ce1—O1^i^	2.349 (6)
Ce1—O3	2.773 (5)
Ce1—O4	2.570 (5)
Ce1—O5^ii^	2.384 (5)
Ce1—O7	2.538 (5)
Ce1—O9	2.538 (5)
Ce1—O11^iii^	2.405 (5)
Ce1—O13	2.560 (5)
Ce2—O2^iv^	2.404 (5)
Ce2—O3	2.594 (5)
Ce2—O6^v^	2.431 (5)
Ce2—O7	2.847 (6)
Ce2—O8	2.657 (6)
Ce2—O10^vi^	2.526 (5)
Ce2—O12^iii^	2.444 (5)
Ce2—O14	2.606 (6)
Ce2—O15	2.646 (6)

Symmetry codes: (i) 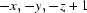; (ii) 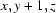; (iii) 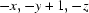; (iv) 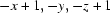; (v) 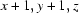; (vi) 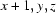.

**Table 2:** Hydrogen-bond geometry (Å, °)

*D*—H⋯*A*	*D*—H	H⋯*A*	*D*⋯*A*	*D*—H⋯*A*
O13—H13*A*⋯O4^vii^	0.82	2.28	2.901 (7)	132
O13—H13*B*⋯N1^viii^	0.81	1.87	2.659 (9)	164
O14—H14*A*⋯N2^vi^	0.89	2.06	2.802 (9)	140
O14—H14*B*⋯O10^vi^	0.88	2.40	3.031 (9)	129
O15—H15*A*⋯O10^vi^	0.82	2.40	2.853 (9)	115
O15—H15*B*⋯N3	0.82	2.00	2.815 (9)	174
C8—H8⋯O12^ix^	0.93	2.43	3.354 (9)	172
C12—H12⋯O9	0.93	2.57	3.354 (9)	143
C15—H15⋯O7	0.93	2.38	3.258 (9)	157

Symmetry codes: (vi) 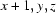; (vii) 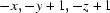; (viii) 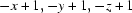; (ix) 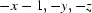.

**Fig. 1.**
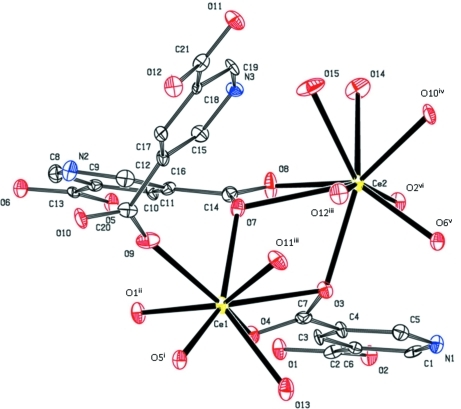
View of the title compound with the atom numbering scheme. Displacement ellipsoids for non-H atoms are drawn at the 50% probability level. H atoms have been omitted for clarity.[Symmetry codes: (i) x, y + 1, z; (ii)-x, -y, -z + 1; (iii) -x, -y + 1, -z; (iv) x + 1, y, z; (v) x + 1, y + 1, z; (vi) -x + 1, -y, -z + 1.]

**Fig. 2.**
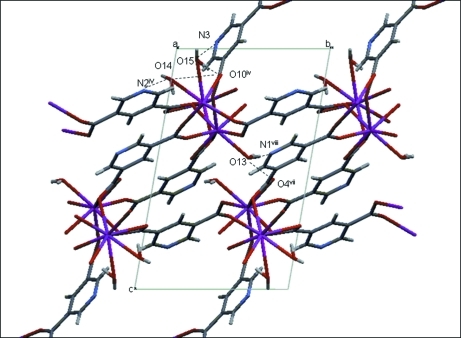
The molecular packing for the title compound. Hydrogen bonds are shown as dashed lines.

## supplementary crystallographic information

### Comment

3,5-PydH_2_ can be easily deprotonated to get a O-donors to N-donor multidentate anion (pyd^2-^), enabling the ligand to act as a bridge to 3 d and/or 4f metal ions (Jia *et al.*, 2006). Some examples of coordination polymer with 3,5-pydH_2_ have been reported (Guo *et al.*, 2009; Li, 2007; Yi *et al.*, 2009).

The asymmetric unit of the title compound contain two independent Ce^III^ atoms, three pyridine-3,5-dicarboxylate(3,5-pyd) ligands and three coordinated water molecules. One Ce^III^ atom is coordinated by nine O atoms from six 3,5-pyd groups and two water molecules, forming a square-face capped square antiprism. Another Ce^III^ atom is coordinated by seven O atoms from six 3,5-pdy groups and one O atom from one water molecule, forming a 4,4'-bicapped trigonal prism. By sharing two carboxylate O atoms *via* a mono atomic bridging mode, two Ce centers are connected into a binuclear unit(Table 1 and Fig.1).

The crystal structure contains an extensive network of classical O—H···O, O—H···N and weak C—H···O hydrogen bonds(full details and symmetry codes are given in Table 2 and Fig. 2).The π···π stacking interactions are also observed, the centroid-centroid distance between the pyridine rings of unbridging 3,5-pyd is 3.509 (4)Å [C*g*5^iii^···C*g*5 (N3/C15—C19)] [symmetry code: -*x*, 1 - *y*, -*z*].

### Experimental

A mixture of Ce(NO_3_)_3_.6H_2_O (0.867 g, 0.2 mmole), 3,5-pydH_2_ (0.495 g, 0.3 mmol) and 10 ml H_2_O was sealed in a 25 ml teflon-lined bomb at 423 K for 3 days and then cooled to room temperature. Colorless crystals were collected (yield 45%, based on Ce(NO_3_)_3_).

### Refinement

Water H atoms were fixed in chemical sensible positions, thier thermal parameters were fixed as 0.08 Å^2^. Other H atoms were positioned geometrically with C—H = 0.93 Å and refined using a riding model, *U*_iso_(H) = 1.2*U*_eq_(C).

### Crystal data

**Table:**

[Ce_2_(C_7_H_3_NO_4_)_3_(H_2_O)_3_]	*Z* = 2
*M**_r_* = 829.60	*F*(000) = 796
Triclinic, *P*1	*D*_x_ = 2.362 Mg m^−^^3^
Hall symbol: -P 1	Mo *K*α radiation, λ = 0.71073 Å
*a* = 8.959 (3) Å	Cell parameters from 1701 reflections
*b* = 9.429 (3) Å	θ = 2.5–25.0°
*c* = 14.582 (4) Å	µ = 3.94 mm^−^^1^
α = 98.115 (6)°	*T* = 295 K
β = 95.501 (6)°	Columnar, colorless
γ = 105.030 (6)°	0.15 × 0.08 × 0.02 mm
*V* = 1166.4 (6) Å^3^	

### Data collection

**Table:**

Bruker SMART CCD area-detector diffractometer	5586 independent reflections
Radiation source: fine-focus sealed tube	2847 reflections with *I* > 2σ(*I*)
graphite	*R*_int_ = 0.090
Detector resolution: 9 pixels mm^-1^	θ_max_ = 28.4°, θ_min_ = 1.4°
φ and ω scans	*h* = −11→11
Absorption correction: multi-scan (*SADABS*; Bruker, 2001)	*k* = −12→12
*T*_min_ = 0.756, *T*_max_ = 0.975	*l* = −18→19
12583 measured reflections	

### Refinement

**Table:**

Refinement on *F*^2^	Primary atom site location: structure-invariant direct methods
Least-squares matrix: full	Secondary atom site location: difference Fourier map
*R*[*F*^2^ > 2σ(*F*^2^)] = 0.041	Hydrogen site location: inferred from neighbouring sites
*wR*(*F*^2^) = 0.076	H-atom parameters not refined
*S* = 0.74	*w* = 1/[σ^2^(*F*_o_^2^) + (0.015*P*)^2^] where *P* = (*F*_o_^2^ + 2*F*_c_^2^)/3
5586 reflections	(Δ/σ)_max_ = 0.002
370 parameters	Δρ_max_ = 1.36 e Å^−^^3^
0 restraints	Δρ_min_ = −1.15 e Å^−^^3^

### Special details

**Table:**

Geometry. Bond distances, angles *etc*. have been calculated using the rounded fractional coordinates. All su's are estimated from the variances of the (full) variance-covariance matrix. The cell e.s.d.'s are taken into account in the estimation of distances, angles and torsion angles
Refinement. Refinement on *F*^2^ for ALL reflections except those flagged by the user for potential systematic errors. Weighted *R*-factors *wR* and all goodnesses of fit *S* are based on *F*^2^, conventional *R*-factors *R* are based on *F*, with *F* set to zero for negative *F*^2^. The observed criterion of *F*^2^ > σ(*F*^2^) is used only for calculating -*R*-factor-obs *etc*. and is not relevant to the choice of reflections for refinement. *R*-factors based on *F*^2^ are statistically about twice as large as those based on *F*, and *R*-factors based on ALL data will be even larger.

### Fractional atomic coordinates and isotropic or equivalent isotropic displacement parameters (Å^2^)

**Table:**

	*x*	*y*	*z*	*U*_iso_*/*U*_eq_	
Ce1	−0.04398 (5)	0.35570 (5)	0.33964 (3)	0.0181 (2)	
Ce2	0.35910 (5)	0.23588 (5)	0.22599 (3)	0.0168 (2)	
O1	0.2438 (6)	−0.1610 (6)	0.6274 (3)	0.0264 (17)	
O2	0.4960 (6)	−0.1188 (6)	0.6760 (3)	0.0237 (17)	
O3	0.2578 (5)	0.3334 (5)	0.3765 (3)	0.0215 (17)	
O4	0.0764 (6)	0.2234 (5)	0.4572 (3)	0.0229 (17)	
O5	−0.1974 (5)	−0.4732 (5)	0.3614 (3)	0.0217 (17)	
O6	−0.4502 (6)	−0.5346 (5)	0.3038 (3)	0.0222 (17)	
O7	0.0313 (6)	0.1587 (5)	0.2310 (3)	0.0222 (17)	
O8	0.1594 (6)	−0.0047 (6)	0.2628 (4)	0.038 (2)	
O9	−0.2542 (6)	0.2878 (6)	0.1987 (3)	0.032 (2)	
O10	−0.4582 (6)	0.3168 (6)	0.1098 (3)	0.0328 (19)	
O11	−0.0774 (6)	0.4892 (6)	−0.2348 (3)	0.032 (2)	
O12	−0.2635 (6)	0.5672 (5)	−0.1697 (3)	0.0273 (17)	
O13	0.1027 (6)	0.5939 (6)	0.4542 (3)	0.0318 (19)	
O14	0.3991 (6)	−0.0037 (6)	0.1307 (4)	0.040 (2)	
O15	0.2259 (7)	0.1451 (7)	0.0501 (4)	0.048 (3)	
N1	0.6058 (7)	0.2665 (7)	0.5638 (4)	0.022 (2)	
N2	−0.3912 (7)	−0.1665 (7)	0.1751 (4)	0.025 (2)	
N3	−0.0159 (7)	0.2521 (7)	−0.0258 (4)	0.024 (2)	
C1	0.5675 (9)	0.1464 (8)	0.6054 (4)	0.021 (3)	
C2	0.4160 (8)	0.0545 (7)	0.5953 (4)	0.014 (2)	
C3	0.3002 (8)	0.0911 (7)	0.5431 (4)	0.016 (3)	
C4	0.3367 (8)	0.2156 (8)	0.4998 (5)	0.016 (3)	
C5	0.4931 (9)	0.2967 (8)	0.5117 (5)	0.022 (3)	
C6	0.3835 (10)	−0.0854 (8)	0.6374 (5)	0.022 (3)	
C7	0.2161 (9)	0.2597 (8)	0.4410 (5)	0.022 (3)	
C8	−0.4043 (9)	−0.2893 (8)	0.2140 (5)	0.026 (3)	
C9	−0.2820 (8)	−0.3157 (8)	0.2669 (4)	0.017 (3)	
C10	−0.1376 (8)	−0.2142 (8)	0.2774 (5)	0.019 (3)	
C11	−0.1195 (8)	−0.0880 (8)	0.2375 (5)	0.016 (2)	
C12	−0.2506 (9)	−0.0689 (8)	0.1882 (5)	0.024 (3)	
C13	−0.3132 (8)	−0.4542 (8)	0.3137 (5)	0.016 (2)	
C14	0.0358 (9)	0.0273 (9)	0.2447 (5)	0.022 (3)	
C15	−0.1092 (9)	0.2494 (8)	0.0402 (5)	0.024 (3)	
C16	−0.2283 (8)	0.3167 (8)	0.0422 (5)	0.019 (3)	
C17	−0.2543 (8)	0.3915 (8)	−0.0296 (5)	0.018 (2)	
C18	−0.1586 (8)	0.3997 (8)	−0.0992 (4)	0.015 (2)	
C19	−0.0426 (8)	0.3287 (8)	−0.0939 (5)	0.020 (3)	
C20	−0.3221 (9)	0.3057 (8)	0.1236 (5)	0.022 (3)	
C21	−0.1691 (9)	0.4919 (8)	−0.1739 (5)	0.021 (3)	
H1	0.64560	0.12380	0.64250	0.0250*	
H3	0.19750	0.03280	0.53670	0.0190*	
H5	0.51990	0.37760	0.48090	0.0260*	
H8	−0.50090	−0.36000	0.20470	0.0310*	
H10	−0.05230	−0.23090	0.31130	0.0220*	
H12	−0.23990	0.01760	0.16270	0.0290*	
H13A	0.05900	0.65940	0.44730	0.0800*	
H13B	0.19210	0.62040	0.44390	0.0800*	
H14A	0.42300	−0.08900	0.13360	0.0800*	
H14B	0.47800	0.05300	0.10900	0.0800*	
H15	−0.09260	0.19820	0.08850	0.0280*	
H15A	0.30020	0.16410	0.02050	0.0800*	
H15B	0.15980	0.18250	0.03010	0.0800*	
H17	−0.33550	0.43610	−0.03150	0.0210*	
H19	0.02160	0.33400	−0.14060	0.0250*	

### Atomic displacement parameters (Å^2^)

**Table:**

	*U*^11^	*U*^22^	*U*^33^	*U*^12^	*U*^13^	*U*^23^
Ce1	0.0143 (3)	0.0185 (3)	0.0231 (3)	0.0042 (2)	0.0029 (2)	0.0094 (2)
Ce2	0.0132 (3)	0.0182 (3)	0.0212 (3)	0.0055 (2)	0.0018 (2)	0.0091 (2)
O1	0.021 (3)	0.023 (3)	0.035 (3)	−0.002 (3)	0.008 (3)	0.017 (3)
O2	0.021 (3)	0.026 (3)	0.028 (3)	0.010 (3)	−0.001 (2)	0.014 (3)
O3	0.020 (3)	0.028 (3)	0.020 (3)	0.007 (2)	0.005 (2)	0.014 (2)
O4	0.018 (3)	0.031 (3)	0.023 (3)	0.009 (3)	0.003 (2)	0.011 (3)
O5	0.017 (3)	0.018 (3)	0.031 (3)	0.007 (2)	−0.002 (2)	0.007 (2)
O6	0.019 (3)	0.021 (3)	0.029 (3)	0.007 (2)	0.004 (2)	0.009 (2)
O7	0.019 (3)	0.016 (3)	0.031 (3)	0.002 (2)	0.002 (2)	0.008 (2)
O8	0.014 (3)	0.027 (4)	0.075 (5)	0.006 (3)	0.002 (3)	0.016 (3)
O9	0.047 (4)	0.034 (4)	0.017 (3)	0.013 (3)	0.002 (3)	0.005 (3)
O10	0.015 (3)	0.057 (4)	0.028 (3)	0.007 (3)	0.008 (3)	0.015 (3)
O11	0.035 (4)	0.044 (4)	0.027 (3)	0.016 (3)	0.013 (3)	0.022 (3)
O12	0.029 (3)	0.023 (3)	0.037 (3)	0.012 (3)	0.006 (3)	0.019 (3)
O13	0.020 (3)	0.025 (3)	0.050 (4)	0.006 (3)	0.003 (3)	0.007 (3)
O14	0.047 (4)	0.022 (4)	0.059 (4)	0.023 (3)	−0.003 (3)	0.011 (3)
O15	0.057 (4)	0.063 (5)	0.033 (4)	0.041 (4)	−0.010 (3)	0.001 (3)
N1	0.021 (4)	0.019 (4)	0.025 (4)	0.001 (3)	0.001 (3)	0.010 (3)
N2	0.021 (4)	0.019 (4)	0.036 (4)	0.008 (3)	−0.001 (3)	0.011 (3)
N3	0.027 (4)	0.031 (4)	0.019 (4)	0.014 (3)	0.004 (3)	0.007 (3)
C1	0.030 (5)	0.025 (5)	0.006 (4)	0.011 (4)	−0.008 (3)	0.001 (3)
C2	0.019 (4)	0.012 (4)	0.013 (4)	0.006 (3)	0.002 (3)	0.002 (3)
C3	0.008 (4)	0.020 (5)	0.021 (4)	0.002 (3)	0.007 (3)	0.007 (3)
C4	0.014 (4)	0.020 (5)	0.017 (4)	0.005 (3)	0.005 (3)	0.012 (3)
C5	0.022 (5)	0.019 (5)	0.024 (5)	0.004 (4)	0.005 (4)	0.005 (4)
C6	0.035 (6)	0.016 (5)	0.015 (4)	0.012 (4)	−0.003 (4)	0.001 (3)
C7	0.024 (5)	0.020 (5)	0.021 (4)	0.012 (4)	0.002 (4)	−0.005 (4)
C8	0.019 (5)	0.023 (5)	0.032 (5)	0.004 (4)	−0.001 (4)	0.002 (4)
C9	0.017 (4)	0.019 (5)	0.015 (4)	0.008 (4)	0.002 (3)	0.001 (3)
C10	0.010 (4)	0.025 (5)	0.024 (4)	0.008 (4)	0.004 (3)	0.007 (4)
C11	0.017 (4)	0.018 (4)	0.012 (4)	0.003 (3)	0.000 (3)	0.002 (3)
C12	0.031 (5)	0.016 (5)	0.027 (5)	0.006 (4)	0.005 (4)	0.009 (4)
C13	0.011 (4)	0.016 (4)	0.020 (4)	0.005 (4)	0.005 (3)	−0.006 (3)
C14	0.023 (5)	0.020 (5)	0.023 (5)	0.005 (4)	0.002 (4)	0.004 (4)
C15	0.031 (5)	0.022 (5)	0.020 (5)	0.005 (4)	0.006 (4)	0.011 (4)
C16	0.015 (4)	0.021 (5)	0.020 (4)	0.000 (4)	0.002 (3)	0.007 (4)
C17	0.015 (4)	0.018 (4)	0.024 (4)	0.012 (3)	0.001 (3)	0.004 (3)
C18	0.012 (4)	0.018 (4)	0.014 (4)	0.001 (3)	0.001 (3)	0.002 (3)
C19	0.014 (4)	0.032 (5)	0.021 (4)	0.011 (4)	0.009 (3)	0.011 (4)
C20	0.033 (5)	0.013 (4)	0.022 (5)	0.004 (4)	0.008 (4)	0.007 (3)
C21	0.026 (5)	0.011 (5)	0.027 (5)	0.004 (4)	−0.001 (4)	0.008 (4)

### Geometric parameters (Å, °)

**Table:**

Ce1—O1^i^	2.349 (6)	O15—H15A	0.8200
Ce1—O3	2.773 (5)	N1—C5	1.319 (10)
Ce1—O4	2.570 (5)	N1—C1	1.342 (9)
Ce1—O5^ii^	2.384 (5)	N2—C8	1.343 (10)
Ce1—O7	2.538 (5)	N2—C12	1.332 (10)
Ce1—O9	2.538 (5)	N3—C15	1.333 (10)
Ce1—O11^iii^	2.405 (5)	N3—C19	1.346 (9)
Ce1—O13	2.560 (5)	C1—C2	1.389 (11)
Ce2—O2^iv^	2.404 (5)	C2—C6	1.505 (10)
Ce2—O3	2.594 (5)	C2—C3	1.372 (10)
Ce2—O6^v^	2.431 (5)	C3—C4	1.391 (10)
Ce2—O7	2.847 (6)	C4—C5	1.392 (11)
Ce2—O8	2.657 (6)	C4—C7	1.494 (11)
Ce2—O10^vi^	2.526 (5)	C8—C9	1.375 (11)
Ce2—O12^iii^	2.444 (5)	C9—C13	1.532 (10)
Ce2—O14	2.606 (6)	C9—C10	1.375 (10)
Ce2—O15	2.646 (6)	C10—C11	1.376 (10)
O1—C6	1.253 (10)	C11—C12	1.383 (11)
O2—C6	1.240 (10)	C11—C14	1.511 (11)
O3—C7	1.275 (9)	C15—C16	1.376 (11)
O4—C7	1.264 (10)	C16—C17	1.377 (10)
O5—C13	1.259 (9)	C16—C20	1.519 (11)
O6—C13	1.247 (9)	C17—C18	1.388 (10)
O7—C14	1.293 (10)	C18—C21	1.497 (10)
O8—C14	1.236 (10)	C18—C19	1.376 (11)
O9—C20	1.255 (9)	C1—H1	0.9300
O10—C20	1.250 (10)	C3—H3	0.9300
O11—C21	1.268 (9)	C5—H5	0.9300
O12—C21	1.238 (10)	C8—H8	0.9300
O13—H13B	0.8100	C10—H10	0.9300
O13—H13A	0.8200	C12—H12	0.9300
O14—H14A	0.8900	C15—H15	0.9300
O14—H14B	0.8800	C17—H17	0.9300
O15—H15B	0.8200	C19—H19	0.9300
			
O3—Ce1—O4	48.97 (15)	Ce1^iii^—O11—C21	139.0 (5)
O3—Ce1—O7	63.21 (15)	Ce2^iii^—O12—C21	147.9 (5)
O3—Ce1—O9	136.42 (15)	Ce1—O13—H13B	109.00
O3—Ce1—O13	74.86 (16)	H13A—O13—H13B	110.00
O3—Ce1—O5^ii^	142.09 (15)	Ce1—O13—H13A	109.00
O1^i^—Ce1—O3	116.29 (16)	H14A—O14—H14B	105.00
O3—Ce1—O11^iii^	81.13 (16)	Ce2—O14—H14A	146.00
O4—Ce1—O7	78.59 (15)	Ce2—O14—H14B	89.00
O4—Ce1—O9	138.31 (16)	Ce2—O15—H15A	103.00
O4—Ce1—O13	84.43 (15)	H15A—O15—H15B	109.00
O4—Ce1—O5^ii^	130.62 (15)	Ce2—O15—H15B	119.00
O1^i^—Ce1—O4	71.74 (17)	C1—N1—C5	117.6 (7)
O4—Ce1—O11^iii^	130.01 (18)	C8—N2—C12	116.8 (7)
O7—Ce1—O9	76.13 (17)	C15—N3—C19	115.7 (7)
O7—Ce1—O13	135.85 (17)	N1—C1—C2	122.7 (7)
O5^ii^—Ce1—O7	148.48 (15)	C1—C2—C3	118.5 (6)
O1^i^—Ce1—O7	86.99 (17)	C1—C2—C6	119.9 (7)
O7—Ce1—O11^iii^	81.55 (16)	C3—C2—C6	121.6 (7)
O9—Ce1—O13	135.94 (17)	C2—C3—C4	119.9 (7)
O5^ii^—Ce1—O9	73.22 (16)	C3—C4—C5	116.8 (7)
O1^i^—Ce1—O9	74.33 (17)	C5—C4—C7	120.8 (7)
O9—Ce1—O11^iii^	77.93 (17)	C3—C4—C7	122.3 (7)
O5^ii^—Ce1—O13	67.88 (17)	N1—C5—C4	124.4 (7)
O1^i^—Ce1—O13	125.59 (16)	O1—C6—C2	116.4 (7)
O11^iii^—Ce1—O13	78.89 (16)	O1—C6—O2	125.6 (7)
O1^i^—Ce1—O5^ii^	91.67 (18)	O2—C6—C2	118.0 (7)
O5^ii^—Ce1—O11^iii^	85.06 (17)	O3—C7—C4	118.9 (7)
O1^i^—Ce1—O11^iii^	151.80 (16)	O3—C7—O4	122.2 (7)
O3—Ce2—O7	61.49 (13)	O4—C7—C4	118.9 (6)
O3—Ce2—O8	76.02 (16)	N2—C8—C9	123.3 (7)
O3—Ce2—O14	142.80 (16)	C8—C9—C10	118.4 (7)
O3—Ce2—O15	132.35 (17)	C8—C9—C13	118.8 (7)
O3—Ce2—O10^vi^	142.82 (16)	C10—C9—C13	122.9 (6)
O3—Ce2—O6^v^	71.96 (15)	C9—C10—C11	119.9 (7)
O3—Ce2—O12^iii^	81.25 (15)	C10—C11—C12	117.5 (7)
O2^iv^—Ce2—O3	86.49 (15)	C12—C11—C14	119.6 (7)
O7—Ce2—O8	47.39 (15)	C10—C11—C14	122.9 (7)
O7—Ce2—O14	103.05 (15)	N2—C12—C11	124.1 (7)
O7—Ce2—O15	73.85 (16)	O5—C13—C9	116.3 (6)
O7—Ce2—O10^vi^	137.07 (15)	O6—C13—C9	117.7 (6)
O6^v^—Ce2—O7	126.71 (16)	O5—C13—O6	126.1 (7)
O7—Ce2—O12^iii^	73.06 (16)	O8—C14—C11	120.8 (7)
O2^iv^—Ce2—O7	115.52 (16)	O7—C14—O8	122.7 (8)
O8—Ce2—O14	69.99 (17)	O7—C14—C11	116.6 (7)
O8—Ce2—O15	86.20 (19)	N3—C15—C16	124.9 (7)
O8—Ce2—O10^vi^	141.16 (17)	C15—C16—C20	118.4 (7)
O6^v^—Ce2—O8	141.34 (16)	C17—C16—C20	123.8 (7)
O8—Ce2—O12^iii^	120.16 (17)	C15—C16—C17	117.8 (7)
O2^iv^—Ce2—O8	72.51 (18)	C16—C17—C18	119.5 (7)
O14—Ce2—O15	60.51 (19)	C17—C18—C19	117.7 (6)
O10^vi^—Ce2—O14	72.40 (17)	C17—C18—C21	122.5 (7)
O6^v^—Ce2—O14	130.08 (18)	C19—C18—C21	119.6 (6)
O12^iii^—Ce2—O14	129.11 (16)	N3—C19—C18	124.4 (7)
O2^iv^—Ce2—O14	69.45 (17)	O9—C20—C16	116.5 (7)
O10^vi^—Ce2—O15	66.93 (18)	O10—C20—C16	117.2 (6)
O6^v^—Ce2—O15	131.76 (17)	O9—C20—O10	126.4 (7)
O12^iii^—Ce2—O15	70.20 (17)	O12—C21—C18	117.4 (7)
O2^iv^—Ce2—O15	129.76 (18)	O11—C21—O12	125.5 (7)
O6^v^—Ce2—O10^vi^	73.31 (16)	O11—C21—C18	117.1 (7)
O10^vi^—Ce2—O12^iii^	77.76 (17)	N1—C1—H1	119.00
O2^iv^—Ce2—O10^vi^	102.93 (17)	C2—C1—H1	119.00
O6^v^—Ce2—O12^iii^	75.75 (16)	C2—C3—H3	120.00
O2^iv^—Ce2—O6^v^	84.34 (17)	C4—C3—H3	120.00
O2^iv^—Ce2—O12^iii^	159.05 (16)	N1—C5—H5	118.00
Ce1^i^—O1—C6	151.9 (5)	C4—C5—H5	118.00
Ce2^iv^—O2—C6	159.8 (5)	N2—C8—H8	118.00
Ce1—O3—Ce2	111.94 (16)	C9—C8—H8	118.00
Ce1—O3—C7	89.4 (4)	C9—C10—H10	120.00
Ce2—O3—C7	126.0 (4)	C11—C10—H10	120.00
Ce1—O4—C7	99.2 (4)	N2—C12—H12	118.00
Ce1^vii^—O5—C13	133.9 (4)	C11—C12—H12	118.00
Ce2^viii^—O6—C13	147.4 (5)	N3—C15—H15	118.00
Ce1—O7—Ce2	111.32 (17)	C16—C15—H15	118.00
Ce1—O7—C14	127.9 (4)	C16—C17—H17	120.00
Ce2—O7—C14	89.2 (5)	C18—C17—H17	120.00
Ce2—O8—C14	99.6 (5)	N3—C19—H19	118.00
Ce1—O9—C20	155.6 (5)	C18—C19—H19	118.00
Ce2^ix^—O10—C20	118.3 (4)		
			
O4—Ce1—O3—Ce2	127.0 (2)	O14—Ce2—O10^vi^—C20^vi^	−71.9 (5)
O4—Ce1—O3—C7	−2.0 (4)	O15—Ce2—O10^vi^—C20^vi^	−136.7 (6)
O7—Ce1—O3—Ce2	28.62 (16)	O3—Ce2—O6^v^—C13^v^	160.2 (9)
O7—Ce1—O3—C7	−100.4 (4)	O7—Ce2—O6^v^—C13^v^	−170.1 (8)
O9—Ce1—O3—Ce2	5.6 (3)	O8—Ce2—O6^v^—C13^v^	124.7 (8)
O9—Ce1—O3—C7	−123.5 (4)	O14—Ce2—O6^v^—C13^v^	15.1 (9)
O13—Ce1—O3—Ce2	−137.1 (2)	O15—Ce2—O6^v^—C13^v^	−68.5 (9)
O13—Ce1—O3—C7	93.8 (4)	O3—Ce2—O12^iii^—C21^iii^	−29.8 (9)
O5^ii^—Ce1—O3—Ce2	−126.3 (2)	O7—Ce2—O12^iii^—C21^iii^	33.0 (9)
O5^ii^—Ce1—O3—C7	104.7 (4)	O8—Ce2—O12^iii^—C21^iii^	38.4 (9)
O1^i^—Ce1—O3—Ce2	100.26 (19)	O14—Ce2—O12^iii^—C21^iii^	126.2 (8)
O1^i^—Ce1—O3—C7	−28.8 (4)	O15—Ce2—O12^iii^—C21^iii^	111.5 (9)
O11^iii^—Ce1—O3—Ce2	−56.34 (18)	O3—Ce2—O2^iv^—C6^iv^	−123.2 (14)
O11^iii^—Ce1—O3—C7	174.6 (4)	O7—Ce2—O2^iv^—C6^iv^	−179.2 (13)
O3—Ce1—O4—C7	2.1 (4)	O8—Ce2—O2^iv^—C6^iv^	160.3 (14)
O7—Ce1—O4—C7	66.4 (4)	O14—Ce2—O2^iv^—C6^iv^	85.7 (14)
O9—Ce1—O4—C7	119.9 (4)	O15—Ce2—O2^iv^—C6^iv^	91.0 (14)
O13—Ce1—O4—C7	−72.7 (4)	Ce1^i^—O1—C6—O2	12.2 (16)
O5^ii^—Ce1—O4—C7	−127.1 (4)	Ce1^i^—O1—C6—C2	−170.3 (7)
O1^i^—Ce1—O4—C7	157.0 (4)	Ce2^iv^—O2—C6—O1	−20.9 (19)
O11^iii^—Ce1—O4—C7	−2.2 (5)	Ce2^iv^—O2—C6—C2	161.7 (10)
O3—Ce1—O7—Ce2	−25.76 (14)	Ce1—O3—C7—O4	3.7 (7)
O3—Ce1—O7—C14	80.9 (6)	Ce1—O3—C7—C4	−175.9 (6)
O4—Ce1—O7—Ce2	−75.35 (18)	Ce2—O3—C7—O4	−113.4 (7)
O4—Ce1—O7—C14	31.3 (6)	Ce2—O3—C7—C4	67.0 (8)
O9—Ce1—O7—Ce2	138.1 (2)	Ce1—O4—C7—O3	−4.0 (7)
O9—Ce1—O7—C14	−115.3 (6)	Ce1—O4—C7—C4	175.6 (6)
O13—Ce1—O7—Ce2	−5.8 (3)	Ce1^vii^—O5—C13—O6	74.3 (9)
O13—Ce1—O7—C14	100.8 (6)	Ce1^vii^—O5—C13—C9	−108.1 (6)
O5^ii^—Ce1—O7—Ce2	124.4 (3)	Ce2^viii^—O6—C13—O5	−78.9 (11)
O5^ii^—Ce1—O7—C14	−129.0 (6)	Ce2^viii^—O6—C13—C9	103.6 (9)
O1^i^—Ce1—O7—Ce2	−147.32 (18)	Ce1—O7—C14—O8	−106.4 (8)
O1^i^—Ce1—O7—C14	−40.7 (6)	Ce1—O7—C14—C11	73.6 (8)
O11^iii^—Ce1—O7—Ce2	58.52 (18)	Ce2—O7—C14—O8	10.4 (7)
O11^iii^—Ce1—O7—C14	165.2 (6)	Ce2—O7—C14—C11	−169.6 (6)
O3—Ce1—O9—C20	−81.5 (12)	Ce2—O8—C14—O7	−11.3 (8)
O4—Ce1—O9—C20	−156.9 (11)	Ce2—O8—C14—C11	168.7 (6)
O7—Ce1—O9—C20	−102.6 (12)	Ce1—O9—C20—O10	−119.1 (11)
O13—Ce1—O9—C20	41.3 (13)	Ce1—O9—C20—C16	60.1 (14)
O5^ii^—Ce1—O9—C20	70.0 (12)	Ce2^ix^—O10—C20—O9	−16.0 (10)
O1^i^—Ce1—O9—C20	166.6 (12)	Ce2^ix^—O10—C20—C16	164.8 (5)
O11^iii^—Ce1—O9—C20	−18.5 (12)	Ce1^iii^—O11—C21—O12	23.5 (12)
O3—Ce1—O5^ii^—C13^ii^	146.4 (6)	Ce1^iii^—O11—C21—C18	−154.5 (5)
O4—Ce1—O5^ii^—C13^ii^	−141.4 (6)	Ce2^iii^—O12—C21—O11	−9.9 (14)
O7—Ce1—O5^ii^—C13^ii^	12.8 (8)	Ce2^iii^—O12—C21—C18	168.1 (5)
O9—Ce1—O5^ii^—C13^ii^	−1.1 (6)	C5—N1—C1—C2	0.2 (10)
O13—Ce1—O5^ii^—C13^ii^	157.7 (6)	C1—N1—C5—C4	2.4 (11)
O3—Ce1—O1^i^—C6^i^	−159.3 (10)	C12—N2—C8—C9	1.3 (11)
O4—Ce1—O1^i^—C6^i^	179.7 (10)	C8—N2—C12—C11	1.0 (11)
O7—Ce1—O1^i^—C6^i^	−101.3 (10)	C19—N3—C15—C16	0.7 (11)
O9—Ce1—O1^i^—C6^i^	−24.9 (10)	C15—N3—C19—C18	−1.0 (11)
O13—Ce1—O1^i^—C6^i^	110.9 (10)	N1—C1—C2—C3	−2.2 (10)
O3—Ce1—O11^iii^—C21^iii^	46.7 (7)	N1—C1—C2—C6	175.0 (6)
O4—Ce1—O11^iii^—C21^iii^	50.0 (8)	C1—C2—C3—C4	1.7 (9)
O7—Ce1—O11^iii^—C21^iii^	−17.4 (7)	C6—C2—C3—C4	−175.4 (6)
O9—Ce1—O11^iii^—C21^iii^	−94.9 (7)	C1—C2—C6—O1	178.4 (6)
O13—Ce1—O11^iii^—C21^iii^	122.9 (7)	C1—C2—C6—O2	−3.9 (10)
O7—Ce2—O3—Ce1	−25.70 (14)	C3—C2—C6—O1	−4.6 (10)
O7—Ce2—O3—C7	80.5 (6)	C3—C2—C6—O2	173.2 (6)
O8—Ce2—O3—Ce1	−74.50 (19)	C2—C3—C4—C5	0.7 (10)
O8—Ce2—O3—C7	31.7 (6)	C2—C3—C4—C7	179.1 (6)
O14—Ce2—O3—Ce1	−98.8 (3)	C3—C4—C5—N1	−2.8 (11)
O14—Ce2—O3—C7	7.4 (7)	C7—C4—C5—N1	178.7 (7)
O15—Ce2—O3—Ce1	−3.1 (3)	C3—C4—C7—O3	−153.3 (7)
O15—Ce2—O3—C7	103.1 (6)	C3—C4—C7—O4	27.1 (10)
O10^vi^—Ce2—O3—Ce1	105.8 (3)	C5—C4—C7—O3	25.1 (10)
O10^vi^—Ce2—O3—C7	−148.0 (5)	C5—C4—C7—O4	−154.5 (7)
O6^v^—Ce2—O3—Ce1	127.5 (2)	N2—C8—C9—C10	−2.7 (11)
O6^v^—Ce2—O3—C7	−126.3 (6)	N2—C8—C9—C13	175.7 (6)
O12^iii^—Ce2—O3—Ce1	49.71 (18)	C8—C9—C10—C11	1.8 (10)
O12^iii^—Ce2—O3—C7	155.9 (6)	C13—C9—C10—C11	−176.5 (7)
O2^iv^—Ce2—O3—Ce1	−147.3 (2)	C8—C9—C13—O5	−180.0 (6)
O2^iv^—Ce2—O3—C7	−41.1 (6)	C8—C9—C13—O6	−2.2 (10)
O3—Ce2—O7—Ce1	28.16 (15)	C10—C9—C13—O5	−1.7 (10)
O3—Ce2—O7—C14	−102.8 (4)	C10—C9—C13—O6	176.1 (7)
O8—Ce2—O7—Ce1	125.4 (3)	C9—C10—C11—C12	0.4 (11)
O8—Ce2—O7—C14	−5.5 (4)	C9—C10—C11—C14	−179.0 (7)
O14—Ce2—O7—Ce1	171.72 (18)	C10—C11—C12—N2	−1.8 (11)
O14—Ce2—O7—C14	40.8 (4)	C14—C11—C12—N2	177.6 (7)
O15—Ce2—O7—Ce1	−134.7 (2)	C10—C11—C14—O7	−159.1 (7)
O15—Ce2—O7—C14	94.4 (4)	C10—C11—C14—O8	20.9 (11)
O10^vi^—Ce2—O7—Ce1	−110.2 (2)	C12—C11—C14—O7	21.5 (10)
O10^vi^—Ce2—O7—C14	118.9 (4)	C12—C11—C14—O8	−158.5 (7)
O6^v^—Ce2—O7—Ce1	−4.2 (2)	N3—C15—C16—C17	0.6 (12)
O6^v^—Ce2—O7—C14	−135.1 (4)	N3—C15—C16—C20	−178.8 (7)
O12^iii^—Ce2—O7—Ce1	−60.96 (17)	C15—C16—C17—C18	−1.6 (11)
O12^iii^—Ce2—O7—C14	168.1 (4)	C20—C16—C17—C18	177.7 (7)
O2^iv^—Ce2—O7—Ce1	98.5 (2)	C15—C16—C20—O9	26.0 (10)
O2^iv^—Ce2—O7—C14	−32.4 (4)	C15—C16—C20—O10	−154.8 (7)
O3—Ce2—O8—C14	69.8 (5)	C17—C16—C20—O9	−153.4 (7)
O7—Ce2—O8—C14	5.9 (4)	C17—C16—C20—O10	25.9 (11)
O14—Ce2—O8—C14	−125.6 (5)	C16—C17—C18—C19	1.4 (11)
O15—Ce2—O8—C14	−65.6 (5)	C16—C17—C18—C21	−173.6 (7)
O10^vi^—Ce2—O8—C14	−110.5 (5)	C17—C18—C19—N3	0.0 (11)
O6^v^—Ce2—O8—C14	104.5 (5)	C21—C18—C19—N3	175.1 (7)
O12^iii^—Ce2—O8—C14	−1.2 (5)	C17—C18—C21—O11	−178.9 (7)
O2^iv^—Ce2—O8—C14	160.5 (5)	C17—C18—C21—O12	2.9 (11)
O3—Ce2—O10^vi^—C20^vi^	92.8 (6)	C19—C18—C21—O11	6.2 (10)
O7—Ce2—O10^vi^—C20^vi^	−162.3 (5)	C19—C18—C21—O12	−172.0 (7)
O8—Ce2—O10^vi^—C20^vi^	−86.8 (6)		

Symmetry codes: (i) −*x*, −*y*, −*z*+1; (ii) *x*, *y*+1, *z*; (iii) −*x*, −*y*+1, −*z*; (iv) −*x*+1, −*y*, −*z*+1; (v) *x*+1, *y*+1, *z*; (vi) *x*+1, *y*, *z*; (vii) *x*, *y*−1, *z*; (viii) *x*−1, *y*−1, *z*; (ix) *x*−1, *y*, *z*.

### Hydrogen-bond geometry (Å, °)

**Table:**

*D*—H···*A*	*D*—H	H···*A*	*D*···*A*	*D*—H···*A*
O13—H13A···O4^x^	0.82	2.28	2.901 (7)	132
O13—H13B···N1^xi^	0.81	1.87	2.659 (9)	164
O14—H14A···N2^vi^	0.89	2.06	2.802 (9)	140
O14—H14B···O10^vi^	0.88	2.40	3.031 (9)	129
O15—H15A···O10^vi^	0.82	2.40	2.853 (9)	115
O15—H15B···N3	0.82	2.00	2.815 (9)	174
C8—H8···O12^xii^	0.93	2.43	3.354 (9)	172
C12—H12···O9	0.93	2.57	3.354 (9)	143
C15—H15···O7	0.93	2.38	3.258 (9)	157

Symmetry codes: (x) −*x*, −*y*+1, −*z*+1; (xi) −*x*+1, −*y*+1, −*z*+1; (vi) *x*+1, *y*, *z*; (xii) −*x*−1, −*y*, −*z*.
